# Morbidity and Mortality after Surgery for Retroperitoneal Sarcoma

**DOI:** 10.3390/curroncol30010039

**Published:** 2022-12-29

**Authors:** Samantha M. Ruff, Valerie P. Grignol, Carlo M. Contreras, Raphael E. Pollock, Joal D. Beane

**Affiliations:** Division of Surgical Oncology, Department of Surgery, The Ohio State University Wexner Medical Center, Columbus, OH 43210, USA

**Keywords:** retroperitoneal sarcoma, surgery, morbidity, mortality

## Abstract

Retroperitoneal sarcoma (RPS) is a rare disease with over 100 histologic types and accounts for 10–15% of all soft tissue sarcomas. Due to the rarity of RPS, sarcoma centers in Europe and North America have created the Transatlantic RPS Working Group (TARPSWG) to study this disease and establish best practices for its management. Current guidelines dictate complete resection of all macro and microscopic disease as the gold standard for patients with RPS. Complete extirpation often requires a multi-visceral resection. In addition, recent evidence suggests that en bloc compartmental resections are associated with reduced rates of local recurrence. However, this approach must be balanced by the potential for added morbidity. Strategies to mitigate postoperative complications include optimization of the patient through improved preoperative nutrition and pre-habilitation therapy, referral to a high-volume sarcoma center, and implementation of enhanced recovery protocols. This review will focus on the factors associated with perioperative complications following surgery for RPS and outline approaches to mitigate poor surgical outcomes in this patient population.

## 1. Introduction

Retroperitoneal sarcoma (RPS) is a rare disease that accounts for 10–15% of soft tissue sarcomas and has over 100 different histology types. The most common subtypes are leiomyosarcoma and liposarcoma [1]. Due to the rarity of RPS, sarcoma centers in Europe and North America have created the Transatlantic RPS Working Group (TARPSWG) to help study this disease and establish best practices for its management. Surgery is the gold standard treatment for RPS because systemic therapies are mostly ineffective. Complete resection of all macro and microscopic disease is consistently shown to be an important predictor of local recurrence and overall survival. In addition, some advocate for a surgical approach that includes resection of the anatomical compartment where the tumor resides. There is no defined compartment in the retroperitoneum, but including natural barriers such as the psoas muscle fascia, vascular adventitia, overlying peritoneum, and organs within 1–2 cm can help to define resection boundaries. This compartmental approach has been associated with a lower local recurrence rate in retrospective studies [2]. Obtaining a negative margin (R0/R1) in patients with RPS can be challenging and is often limited by anatomic constraints of critical organ involvement such as major blood vessels and vital organ involvement. For this reason, local recurrences are more frequent in patients with RPS than in those with soft tissue sarcoma of the extremity. In 2015, the TARPSWG concluded that RPS are best managed by a multidisciplinary team and that the best oncologic outcome is associated with a curative surgery at the time of initial presentation [3].

Historically, there has been a concern that a more radical resection would result in increased surgical complications [3]. In a retrospective study of 1007 patients who underwent surgical resection for RPS, 16.4% of patients had a major postoperative adverse event and 1.8% died within 30 days [4]. While comparing individual metrics of surgical quality such as morbidity, readmission rate, or mortality is useful, there are significant limitations, and this may oversimplify the patient experience. As such, these individual metrics often do not reflect the overall quality of care a surgical patient receives. To address this limitation, a composite metric known as a textbook outcome was created. As an all-or-none metric composed of multiple, individual measures of quality, textbook outcome aims to ensure all desired perioperative outcomes are met, thereby providing a more global picture of the overall quality of care a patient receives [5]. When analyzed retrospectively in 627 patients who underwent resection for RPS, those who achieved a textbook outcome experienced a median overall survival twice as long as those who failed to achieve a textbook outcome. This study suggests an association between perioperative complications and oncologic outcome [5]. This highlights the importance of achieving a balance between an aggressive surgical approach with the potential morbidity/mortality to achieve an optimal oncologic outcome. This review will focus on the factors that contribute to perioperative complications after surgery for RPS and examine the interventions available to reduce morbidity and mortality.

## 2. Post-Operative Morbidity and Mortality of RPS Operations

### 2.1. Overall Morbidity and Mortality

Given the heterogeneity and diverse anatomic quadrants from which RPS can arise, the operation needed to achieve complete removal varies significantly amongst patients. Accordingly, there is a wide range of potential complications depending on the organs resected and surgery performed. An analysis from the American College of Surgeons National Surgical Quality Improvement Program (NSQIP) of 564 patients found that there was no significant difference in overall morbidity, severe morbidity, or mortality between patients who underwent multi-visceral resection compared to those who did not. However, when looking at individual complications, the multi-visceral resected group had a higher rate of deep incisional surgical site infection and sepsis. This study found that longer operative time, lower serum albumin, leukocytosis, and tumor size ≥ 10 cm were associated with increased 30-day overall morbidity [6]. In a single institution study of 58 patients who underwent resection for RPS, postoperative morbidity (Clavien–Dindo ≥ 3) and mortality was 24.1% and 1.3%, respectively [7]. 

Overall morbidity and mortality rates are consistent among most published series. In a retrospective study of 1007 patients who underwent surgical resection for RPS, 16.4% of patients had a major postoperative adverse event (the most common were bleeding/hematoma and anastomotic leak) and 1.8% died within 30 days. Short-term surgical outcomes in this study following compartmental resection of RPS were comparable to historical controls, including those with less radical resections. The authors attributed this, in part, to the fact that many patients were treated at high-volume sarcoma referral centers with extensive experience in preoperative patient optimization, intraoperative decision making, and postoperative management. Importantly, they found that adverse events did not have an impact on overall survival or the rate of local recurrence/distant metastases [4]. While this study showed that adverse events did not impact overall survival, a recently published study by Tirotta et al. may suggest otherwise. The authors used a composite score (comprehensive complication index (CCI)) to assess the burden of multiple complications in 191 patients at a single institution over a 10-year period. They found that an increased CCI (>20.9, equitable to at least a Clavien–Dindo grade 2 complication) was associated with worse overall survival (HR 2.31, *p* = 0.004). At one year, mortality was <10%, approximately 20%, and almost 50% for patients with a CCI of 20.9, 40, and 60, respectively. However, it is important to interpret this data in the context of two key points. First, all Clavien–Dindo grades were included and therefore 82.9% of patients were considered to have developed a complication. Second, there was no association between the increased CCI and local recurrence or development of distant metastases [8]. 

### 2.2. Impact of Vascular Resections on Post-Operative Outcomes

Leiomyosarcomas are one of the most common forms of RPS and arise from blood vessels, in the retroperitoneum most (75%) arise from the inferior vena cava (IVC) [9]. In addition, other RPS can also encase the vessel making it either too dangerous to separate from the vessel wall or result in leaving macroscopic disease that would quickly recur. Historically these patients were treated nonoperatively but given advancements in operative techniques and vascular reconstruction many patients are now offered surgery. As a result, the impact of vascular resection and reconstruction on operative morbidity and mortality has become a focus in predicting quality outcomes following surgery for RPS.

In one international cohort of patients, vascular resection or pancreaticoduodenectomy was associated with the greatest perioperative morbidity [4]. A single-center study of 17 patients demonstrated that vascular reconstructions can be safely undertaken with an acceptable perioperative complication rate. This study also included an extensive literature review of 110 patients. In the study of this institution’s 17 patients, 41% had postoperative complications and in the literature review cohort (110 patients), 30% had perioperative complications. Of the 110 patients reviewed in the literature, the IVC was the most commonly reconstructed vessel and 87% of the time performed with an interposition graft. On the final pathology of the 17 patients, half of these patients had an invasion of the tumor into the wall of the resected vessel, or the tumor was arising from the vessel wall. These findings suggest that dissecting these tumors from the vessel wall may not result in the best oncologic outcome [10]. 

**Figure 1:**
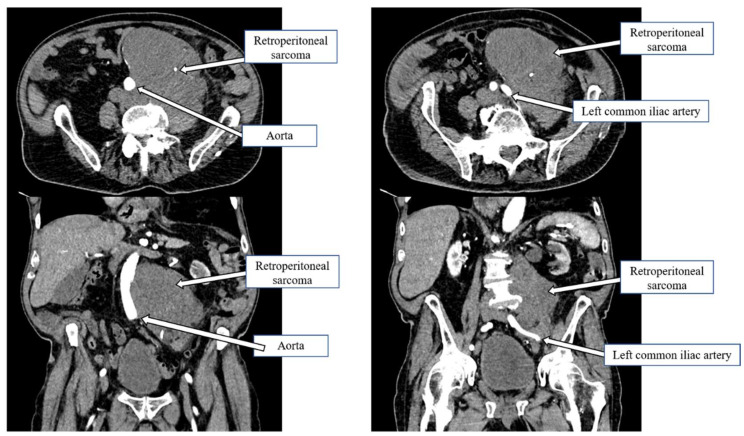
Retroperitoneal sarcoma wrapped around the infrarenal aorta down to the iliac bifurcation.

**Figure 2:**
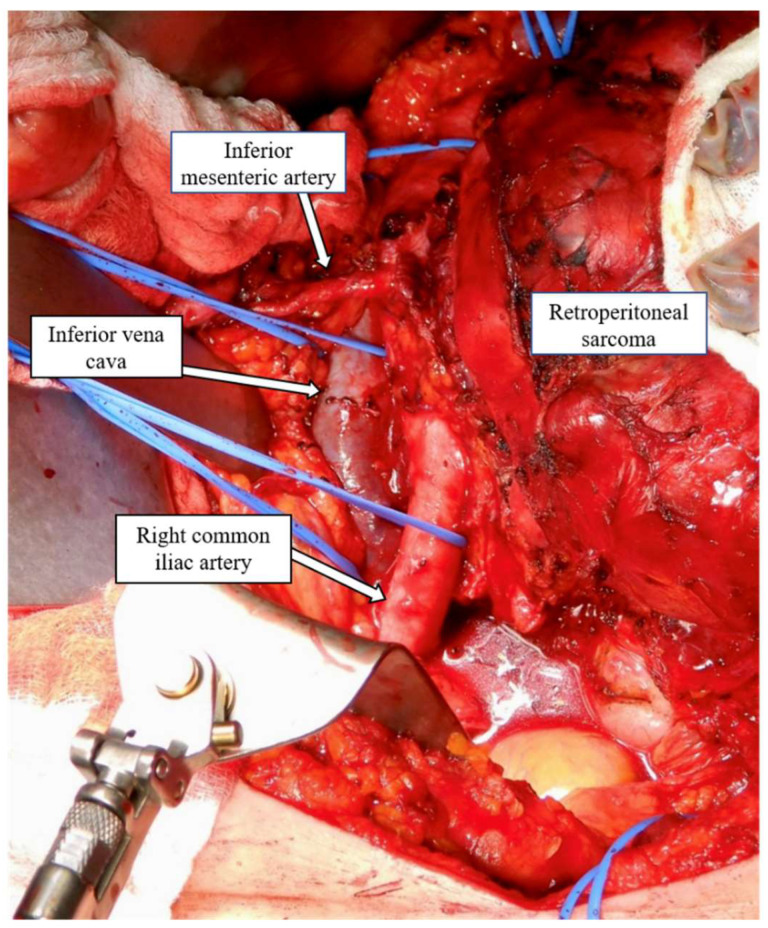
Retroperitoneal sarcoma encasing the infrarenal aorta.

While vascular resection and reconstruction may be associated with a superior oncologic outcome, this must be balanced with the potential for added morbidity. In a study of 425 patients with liposarcomas at a single institution over an 18-year period, 5% required a vascular resection. Vascular resection was associated with longer operative time and had a higher rate of major complications (54% compared to 25%, *p* = 0.002). Patients requiring vascular resection had a lower 5-year OS (60% vs. 81%, *p* = 0.05) and a higher incidence of local and distant recurrence at 5 years (local 45% vs. 24%, *p* = 0.05, distant: 20% vs. 0%, *p* = 0.04). The association between vascular involvement of liposarcoma and the risk of recurrence may be due to more aggressive biology. This should be discussed with the patient in the preoperative setting given the associated risks [11]. However, recent reports have demonstrated the safety and feasibility of major vascular resections at the time of surgery for RPS. A study of 67 patients who underwent IVC or iliac vein resection/reconstruction for RPS demonstrated only 22.4% of patients had a Clavien–Dindo grade ≥ 3 complications within 60 days of surgery. Of the 32 patients who required IVC reconstruction, there was 100% patency of the IVC polytetrafluoroethylene grafts and 76.7% patency of the IVC banked venous homografts at 5 years. Overall, complication rates (22%) were comparable to patients who underwent multi-visceral resection without vascular involvement in the literature. This study demonstrates not only the short-term complications but that long-term patency can be maintained [12]. Similarly, Schwarzbach et al. evaluated 141 patients with RPS with vascular involvement. They found an overall complication rate of 36% and strong long-term patency rates (88.9% for arterial patency and 93.8% for venous patency at 19.3 months) [13]. These studies demonstrate that vascular reconstructions can be performed in appropriately selected patients for the purpose of a better oncologic resection without increasing the perioperative complication rate (Figure 1 and Figure 2).

### 2.3. Nephrectomy in RPS

Given the anatomic location of RPS, a nephrectomy is one of the more common organs resected en bloc with the tumor. In the TARPSWG study of 1007 patients, 54.8% of patients underwent a nephrectomy [4]. In addition to the short-term outcomes, this may also affect long term morbidity and mortality if the patient develops chronic kidney disease. A retrospective study of 54 patients who underwent nephrectomy as part of their surgery demonstrated that the glomerular filtration rate (GFR) initially decreased postoperatively from a median of 85 mL/min to 44 mL/min. At a median follow up of 50 months, the GFR had increased to a median of 62 mL/min, but was not back to preoperative baseline. In this cohort, 51% of the patients with preoperative chronic kidney disease (CKD) stage 1–2 (eGFR ≥ 60 mL/min) had preserved postoperative GFR, but 49% progressed to CKD stage 3 (GFR 30–50 mL/min). It is important to note that at four year follow up no patients had progressed to end stage renal disease. Risk factors for progression to stage 3 were age and preoperative GFR [14]. Another retrospective study of 113 patients who underwent nephrectomy found similar results (preoperative GFR went from 89.2 mL/min on average to 46 mL/min before rebounding to 58.1 mL/min at a median follow-up of 20 months). Similarly, half of the patients progressed from CKD stage 1–2 to CKD stage 3 [15]. However, in another study of 95 patients (64 underwent nephrectomy), there was no difference in the change of median creatinine levels between patients who did or did not undergo a nephrectomy as part of their resection after adjusting for age and baseline creatinine levels (*p* = 0.170). For all patients, creatinine concentration was within 1.5 times the upper reference limit at the 4–6 month postoperative visit [16]. 

More recently, a study from the US Sarcoma Collaborative created a matched cohort of 411 patients who did not undergo a nephrectomy with 108 patients who had a nephrectomy during RPS resection. The patients who underwent a nephrectomy had a higher rate of postoperative acute kidney injury (AKI), but no patients required dialysis. However, this study also showed that there was a significantly high recurrence rate in patients who did not undergo a nephrectomy [17]. Cho et al. evaluated 114 patients (65 with nephrectomy compared to 49 without nephrectomy) and demonstrated that even though the patients in the nephrectomy group had a statistically significant decrease in GFR between pre and postoperative values compared to the no-nephrectomy group (*p* = 0.001), this number stabilized within 6 weeks of surgery. No patients progressed to end-stage renal disease [18]. 

Collectively these studies demonstrate that nephrectomy does confer an increased risk of postoperative acute kidney injury and worsening chronic renal function. However, despite the risk of short- or long-term complications, not performing a nephrectomy when indicated increases the risk of local recurrence. An accurate preoperative assessment of kidney function and renal scintigraphy scans should be included in select patients during the preoperative evaluation to appropriately risk stratify and counsel patients.

### 2.4. Impact of Bowel Resection on Post-Operative Outcomes

In one of the largest retrospective series published to date, 64% of patients required a bowel resection as part of their surgery for RPS (*n* = 645/1007). Of patients who underwent bowel resection, colectomy was performed most frequently (90%) [4]. In another study of 118 patients, the colon was the second most common organ resected (17%) after a nephrectomy (19%). Importantly, this study also found that 25% of resected colons had evidence of microscopic invasion. This reinforces that compartmental resections in patients with RPS is appropriate and may improve oncologic outcomes [19]. In an analysis of factors associated with receiving a textbook outcome (defined as R0/R1 resection, discharge home without requiring a blood transfusion, reoperation, or grade ≥ 2 complication, hospital-stay >50th percentile, or 90-day readmission/mortality), the authors found that a left colectomy or low anterior resection was associated with not achieving a textbook outcome (OR 0.42, *p* = 0.03) [5]. In a study of 249 patients who underwent surgery for RPS, 18% of patients had a complication that required an invasive procedure as part of the management. The most common complication was an anastomotic leak, with over half resulting from colonic anastomoses. However, on multivariable analysis, colon resection was not associated with increased morbidity compared to other organs. It is also important to note that surgical complications in this study did not influence the oncological outcome on multivariable analysis [20]. 

### 2.5. Impact of Other Organ Resections on Post-Operative Outcomes

Some patients will also require resection of other organs, such as the adrenal gland, duodenum, pancreas, or uterus/adnexa. Given that these resections are less common, it can be more difficult to assess organ-specific post-operative complications and their impact on a patient’s outcome and prognosis. In a study of 50 patients who underwent en bloc resection of a RPS and adrenal gland, 64% of patients had adrenal insufficiency in the early postoperative period and 38.5% on long-term follow-up (diagnosed through an elevated ACTH 4 months after surgery). One-third of patients did not develop adrenal insufficiency during the study period, 28.2% had early transient adrenal insufficiency that resolved at long-term follow-up, 23.5% of patients developed late adrenal insufficiency first diagnosed at long-term follow-up, and 28.2% had persistent adrenal insufficiency from the early to late postoperative period. Early adrenal insufficiency did not correlate with admission to the intensive care unit, need for vasoactive medications, or postoperative morbidity and mortality. However, it is important to note that while patients may have some adrenal insufficiency during the postoperative period that can persist for months after surgery, the impact is often inconsequential as patients did not require corticosteroid replacement therapy. However, if adrenalectomy is required, screening high-risk patients for pre-existing subclinical adrenal insufficiency is prudent [21]. 

One of the most common complications following pancreatectomy is the development of postoperative pancreatic fistula. In a study of 2068 patients who underwent primary RPS resection, 29 patients (1.4%) required a pancreatoduodenectomy. In 84% of these patients, there was microscopic invasion on the specimen (duodenum or pancreas). Ten patients (34%) had a major complication and eight of those patients developed a clinically significant pancreatic leak [22]. Given the small sample size, it is unclear what effect a pancreatic leak may have on oncologic outcomes. The study demonstrated that 66% of patients had a recurrence of disease at a median follow-up of 4.8 years. However, this may just reflect that patients who require a pancreatoduodenectomy for RPS resection have more aggressive diseases [22]. In a separate TARPSWG study of 1007 patients, pancreatoduodenectomy was an independent risk factor for postoperative morbidity [4]. A more recent study published in 2020 evaluated 50 patients with primary or recurrent RPS who underwent surgery and required either a distal pancreatectomy or pancreatoduodenectomy. The postoperative Clavien–Dindo grade 3/4 morbidity rate was 28%. Ten patients had a pancreatic fistula (12 grade A, 6 grade B, 1 grade C) [23]. 

## 3. Risk Factors for Post Operative Morbidity and Mortality

### 3.1. Patient-Related Factors

The most cited patient factors associated with perioperative morbidity and mortality are age and pre-existing co-morbidities. A study from the TARPSWG found that age > 65 was associated with increased postoperative morbidity (OR 1.5, CI 1.06–2.13, *p* = 0.031) [4]. In a retrospective study from the United Kingdom of 392 patients who underwent RPS resection, the only significant factor associated with 30-day mortality was age > 75 [15]. Neither of these trials included co-morbidities as potential variables that may impact postoperative outcomes in their analysis. However, a study of 692 patients found that increasing age was associated with higher mortality at one year on multivariable analysis. In addition, they evaluated whether incorporating a co-morbidity score or ECOG performance status into the analysis changed the association between age and survival. Even after accounting for these variables, the authors found that age was still an independent risk factor for 1-year mortality. On subgroup analysis of patients who died within a year of surgery, a higher percentage were secondary to post-operative complications in the older age groups (0% in the <55 and 55–64 age groups versus 22% in the 65–74 group and 28% in the ≥75 group) [24]. Other studies have found that age is not associated with increased morbidity or mortality after RPS resection. A retrospective study of the US Sarcoma Collaborative demonstrated that there was no difference in total complications, major complications, or mortality between patients ≥70 or <70 years old. Elderly patients were more likely to be discharged to a skilled nursing or rehabilitation facility. There was no difference in 3-year disease-free survival between the two groups, but the elderly patients did have a lower 3-year disease-specific survival (60% versus 76% in the <70 years old group versus ≥ 70, *p* < 0.001) [15]. It is important to recognize that these studies all used a different age cut-off for the “elderly” population and none of these studies evaluated age as a continuous variable, both of which can create bias.

The discrepancy between these retrospective studies and in what variables are included in analysis, suggests that age should not be the sole contra-indication to performing an aggressive resection. As patients age, they have less physiologic reserve. However, other factors, such as co-morbidities and the subsequent effects of those co-morbidities that accumulate with time, must also be considered. In an analysis of 385 patients, patients older than 65 had a higher rate of non-operative management (41.8% compared to 12% in the <65 cohort) even though there was a similar number of patients with resectable tumors in the elderly versus non-elderly cohorts. The authors attributed this difference in treatment strategy to differences in comorbidities and patient preference. This study demonstrated that patients > 65 had higher perioperative morbidity, but no difference in perioperative mortality or oncologic outcomes [15]. Finally, in a study of 191 patients between 2008–2019, Tirotta et al. found that the cumulative burden of complications portrayed as the CCI was more strongly associated with a longer hospital length of stay than the Clavien–Dindo complication grade [25].

Textbook outcome is a composite metric aimed at ensuring all desired perioperative outcomes are met to accurately measure the overall quality of care. In a study of 627 patients, one of the most common reasons for not achieving a textbook outcome was postoperative complications (33.2%). In this study, this was defined as the absence of R2 resection, grade ≥ 2 postoperative complications, transfusion of packed red blood cells perioperatively or postoperatively, reoperation, hospital length stay ≥50th percentile, hospital readmission within 90 days, non-home discharge, and any mortality within 90 days. On univariate analysis, co-morbidities associated with not achieving a textbook outcome were the presence of hypertension, prior cardiac event, and dyspnea. On multivariable analysis, ASA class 3 or 4 and prior cardiac event were associated with not achieving a textbook outcome [5]. Boyle et al. characterized body composition among 95 patients with RPS and trunk sarcomas and demonstrated that increased intramuscular adiposity was associated with increased wound infections and major complications [26]. Finally, a retrospective study of 40 patients who underwent a RPS operation demonstrated that malnourishment was associated with a longer hospital stay and a higher number of perioperative complications [27]. 

### 3.2. Treatment-Related Factors

There are few studies that evaluate the role of chemotherapy in RPS and therefore it is difficult to evaluate how its use may affect the morbidity and mortality of operating on RPS [28]. On the other hand, the use of neoadjuvant radiation therapy for RPS has increased over the past few decades. There are multiple potential benefits to neoadjuvant radiation, but little level 1 evidence to support it. Neoadjuvant radiation for RPS may improve tumor resectability and margin status. Radiation is most effective in an oxygen-rich environment. This means, that it may be more effective when the tumor vasculature is intact. The tumor will also displace normal tissue and protect nearby organs from the radiation field [29]. According to the NCCN guidelines, neoadjuvant radiation for RPS should be considered in patients with high risk of local recurrence [30]. This recommendation is controversial due to the paucity of data to support it. There are small prospective and retrospective studies that showed an acceptable 5-year recurrence-free survival, disease-free survival, and overall survival in patients with intermediate or high-grade RPS after neoadjuvant radiation and an R0 or R1 resection [31,32]. However, the STRASS trial, a randomized phase 3 study of patients with RPS, compared those who had preoperative radiation and surgery versus surgery alone. This trial did not demonstrate a difference in recurrence-free survival between the two groups [33]. Despite this, post-trial analysis of the STRASS data suggested that neoadjuvant radiation therapy may be favorable in patients with well-differentiated liposarcoma.

Given the controversy regarding whether neoadjuvant radiation therapy provides an oncologic benefit, it is important to ensure that neoadjuvant radiation does not confer any increased risk of perioperative complications. Several single-institution studies have attempted to determine perioperative morbidity, but due to the rarity of this disease, the studies are often underpowered or lack granular data [34,35,36]. The TARPSWG did not see an association between neoadjuvant radiation and postoperative morbidity [4]. A benefit of large national databases is that data can be pooled to help compensate for the bias inherent to retrospective studies. Utilizing the NSQIP database, Nussbaum et al. used propensity score matching to compare 30-day perioperative complications between patients who received neoadjuvant radiation therapy and those who only underwent surgery. There was no difference in mortality, major complications, overall complications, early reoperation, or length of stay between the two groups [29]. A similar NSQIP study used multivariable analysis to demonstrate that there was no association between neoadjuvant radiation therapy and postoperative morbidity and mortality [37].

In addition to oncologic and postoperative outcomes, the final question about neoadjuvant radiation is when is the safest time to operate. Optimal timing may help to maximize tumor response, optimize tissue planes (given tissue edema from radiation), and minimize wound complications. Louie et al. evaluated their extremity and RPS surgical experience over a 20-year study period to see if the length of time (<6, 6–8, 8–10, or >10 weeks) between neoadjuvant external beam radiation therapy (EBRT) and surgery affected perioperative outcomes. They found that even though the time interval did not affect oncologic outcomes, a longer interval did affect the rate of postoperative complications. The overall complication rate in patients was 28% with 63% of them being wound complications. On multivariable analysis, they found that surgery >6 weeks after completion of EBRT was associated with increased perioperative complications. Other factors associated with perioperative morbidity were retroperitoneal sarcoma (compared to extremity) and increasing Charlson comorbidity index [38].

### 3.3. Intraoperative Factors

Given the complexity of these resections, many studies have focused on operative factors that may contribute to increased morbidity and mortality after RPS resection. Across multiple retrospective studies, the need for transfusion and the resected organ score are two variables that are consistently associated with complications following surgery [4,39,40]. Transfusion requirements’ association with postoperative complications is likely multifactorial. Receipt of a transfusion may be an indicator of elevated blood loss from a challenging operation or an operation that involved vasculature reconstruction. Another possibility is that the blood is given as a response to a postoperative hematoma, which means the transfusion is in response to the complication rather than the predictor of a complication. Multiple studies have shown the association between transfusion requirement and perioperative morbidity and mortality [4]. One retrospective study of 192 patients found that perioperative blood transfusions were associated with severe postoperative complications (≥3 on Clavien–Dindo scale). It was also noted though that preoperative anemia and blood loss >1000 mL were both predictive of receiving perioperative blood transfusions and there was a correlation between a higher resected organ score and how many units of blood were transfused [39].

Given that blood transfusions are often just indicative of a more complex operation, many studies have started to focus on scoring the operation based on how many and which organs were resected. The TARPSWG study weighted different organs from 0–2 based on their risk of postoperative morbidity. They found that there was an association between resected organ score and operative timing and on multivariable analysis resected organ score was associated with severe adverse events (Clavien–Dindo ≥ 3) [4]. This method has been applied in other studies as well. In a study of 249 patients with RPS, Bonvalot et al. found an increase in morbidity for patients who had more than three organs resected [20].

## 4. Methods to Improve Perioperative Morbidity and Mortality after RPS Operations

### 4.1. High-Volume Centers for RPS

Rare cancers are difficult to treat because there is often little consensus on the best treatment plan due to the paucity of data. In the past few decades, there has been a concerted effort to centralize the treatment of rare cancers in centers throughout the country and the world. This is beneficial for two reasons. First, in theory, patients receive the best possible care from the leading experts in the field who are up to date on the available data. Perhaps even more crucial, is that the staff who care for the patient in the pre, intra, and postoperative setting are well versed in the rare disease, its common complications, and what a normal hospital course looks like. This has been ascribed as the “experience effect” [40]. The nuances of caring for these patients are more routine and effective on an individual level and on a systems level. High-volume centers have been shown to have improved short- and long-term outcomes for multiple types of cancers and surgeries [41,42,43,44]. This is also true for patients with retroperitoneal sarcoma. Given the rarity and complexity of RPS, the TARPSWG recommends that patients be managed by a multidisciplinary team in a specialized sarcoma referral center. TARPSWG recommends that a sarcoma referral center resect a minimum of 10–20 RPS cases per year, include a multidisciplinary team with a surgeon, radiologist, pathologist, medical oncologist, and radiation oncologist and that every effort should be made to include eligible patients in clinical trials and contribute RPS cases to a prospective database [45]. They have demonstrated that there is better adherence to clinical practice guidelines, improved early postoperative morbidity, reduced risk of postoperative mortality, and improved long-term overall survival [4]. In 2017, the Sarcoma Policy Checklist was created by a European multidisciplinary group that recommended each country have one designated and accredited center for sarcoma referral to ensure appropriate, specialized care for this rare malignancy [46].

The benefit of treating patients at a high-volume center has been demonstrated in large retrospective studies. The National Cancer Database was queried from 1998–2011 and evaluated 6950 patients who underwent primary resection of RPS and found that 90% of them were treated at low volume centers (defined as treating on average <10 cases/year). This study demonstrated that patients treated at low-volume centers had higher 30-day readmission rates (3.4% compared to 1.8%, *p* < 0.0001), 30-day mortality (3.1% versus 1.9%, *p* = 0.004), and 90-day mortality (5.7% versus 3.2%, *p* = 0.007). In addition, after controlling for other variables, patients treated at high-volume facilities had a reduced risk of death [47]. A second NCDB study identified 8721 surgically treated RPS patients between 2004–2015 and showed that overall mortality risk was reduced by 4% per additional case up to a threshold of 13 cases/year. A comparison of survival between hospitals with <13 vs. >13 cases per year was 94 versus 139 months, respectively. This group surveyed members of the TARPSWG and found that 29% of survey respondents felt that >30 cases/year should be the cut-off for a high-volume center. Yet, after seeing the results from the aforementioned NCDB study, 71.4% of these respondents chose a lower cutoff value. In addition, 39.6% of respondents cited 1–2% as an acceptable 90-day mortality. Using the NCDB, this was achieved with a minimum of 13 cases/year as the cutoff [48]. 

In 2009, hospitals within the Merseyside network in the UK consolidated their RPS care. They demonstrated that centralizing their patients’ care led to an increase in resection rates, complex multi-visceral resections (without compromising R0/R1 resection rates), improved perioperative mortality, and overall survival [49]. In Switzerland, a retrospective study analyzed patients with RPS between 2005–2015 comparing those treated in sarcoma referral centers to those treated in non-sarcoma centers. In concordance with international guidelines, they found an increase in the number of patients being treated at the sarcoma referral centers over time. In addition, there was a higher complication rate in patients treated at non-sarcoma centers (55%) compared to patients treated at sarcoma centers (40%). The in-hospital surgical mortality rate was 1.4% at sarcoma centers compared to 4% at non-sarcoma centers [50].

While centralizing care of rare disease to high volume centers improves short term and oncologic outcomes, there are some associated challenges. The main challenge lies in ensuring patients have access to these referral centers. From a practical standpoint, not every patient can travel to a specialized center, either due to time, availability of close support system, or financial restraints. More work is still needed to address this obstacle [51]. 

### 4.2. Enhanced Recovery Protocol for RPS

Evidence-based, standardized protocols for perioperative care, commonly referred to as enhanced recovery after surgery (ERAS) protocols, have become implemented as part of the routine postoperative care for nearly all surgical procedures. ERAS protocols were designed to standardize the postoperative management of patients based on available evidence for best practices to help minimize complications. The improvement in outcomes is driven by order sets whereby multiple facets (pain management, physical therapy, nutrition, fluid management) are standardized amongst patients undergoing a specific operation. While no single intervention alone may alter the patient’s course, the sum of incremental improvements when combined has dramatically altered postoperative outcomes. The success of these programs relies on teamwork, adherence, continuous re-evaluation of outcomes to make necessary adjustments, and updating protocols based on new evidence. Attributable to a reduction in hospital stay and postoperative morbidity, these protocols also result in a cost–benefit for the hospital. As a result, these protocols represent a paradigm shift in how surgical care is delivered [52]. Studies in other cancer types have demonstrated a correlation between adherence to ERAS protocols and improved oncologic outcomes. While it is not clear if this is a true cause-and-effect relationship, it is possible that compliance with these protocols may lead to decreased perioperative morbidity and therefore optimize timing and tolerance of adjuvant therapies. In addition, it has been shown that patients in an ERAS setting have better preserved immune function. This could indirectly influence tumor recurrence or metastases [53]. 

A benefit of standardizing guidelines for the treatment of patients who undergo surgery for RPS is that evidence-based enhanced recovery protocols can be developed and implemented. In 2015, enhanced recovery after surgery (ERAS) protocol was implemented at the Dana-Farber Cancer Institute in Boston. This study compared patients who underwent surgery for any sarcoma (trunk, retroperitoneal, head/neck, extremity, abdominal) before and after the implementation of the protocol. The ERAS cohort had fewer wound dehiscence (0.9% versus 13.1% in the non-ERAS cohort) and postoperative ileus/bowel obstructions (9% versus 16.9% in the non-ERAS cohort). The ERAS cohort also had a shorter median length of stay (5 days versus 6 days in the non-ERAS cohort). A sub-analysis of retroperitoneal sarcomas (36 patients in each cohort) was performed. The ERAS cohort had fewer readmissions (3% versus 28% in the non-ERAS cohort), wound dehiscence (0% versus 22% in the non-ERAS cohort), and ileus/bowel obstruction (11% versus 42% in the non-ERAS cohort). In addition, the ERAS cohort had a shorter median length of stay (8 days versus 14 days in the non-ERAS cohort) [54]. Unfortunately, there is very limited data on ERAS protocols in the RPS patient population and this one study is not specific to RPS, but to all soft tissue sarcomas. Centralizing care of RPS to high-volume centers is key to reducing postoperative morbidity and mortality. As a community, we should be working to strategically establish these centers throughout the country where there is limited access to specialized sarcoma care. In addition, it will be important to continue to work with the primary care doctors who likely see these patients first and curate resources to help patients get to a specialty center. 

### 4.3. Pre-Operative Nutrition

Several studies have demonstrated that patients with pre-operative malnutrition have a longer postoperative length of stay and/or higher complication rates [27]. As a result, optimizing pre- and postoperative nutrition has become a key component of ERAS protocols. This is especially important for patients with RPS given that surgery often requires multi-visceral resections. A retrospective study of patients who underwent surgery for retroperitoneal liposarcoma demonstrated that pre-operative malnutrition was associated with longer hospitalization and postoperative morbidity [27]. A prospective study found that 46% of their patients with RPS had preoperative protein energetic malnutrition. The authors placed these patients on an oral support regimen to improve pre-operative nutrition. However, at the time of surgery, 38% of patients still qualified as malnourished. The authors did find an association between preoperative malnutrition and increased risk of a Clavien–Dindo grade 3 or 4 complications [55]. Nutrition is clearly a risk factor for increased morbidity, but an optimized nutrition intervention still needs to be prospectively tested in patients with RPS.

## 5. Risk Associated with Recurrent Operations for RPS

### Recurrent RPS Operations

Despite a shift towards radical resections of RPS, studies report a range of 10–29% local recurrence rate that often require repeat operations [28]. However, there is little data on whether there is increased morbidity and mortality after re-resections. The violation of the original anatomic planes during the initial operation and the potential for extensive lysis of adhesions could make the surgery more difficult. In addition, patients who have recurred sometimes undergo chemotherapy and/or radiation therapy, which may affect the perioperative complication rates. In another study of 681 patients who underwent surgery for recurrent RPS, 16% of patients had a major complication after surgery and 0.4% had a 90-day mortality rate. The most common complication was an anastomotic leak. Only blood transfusions were associated with having a major complication in the multivariable model. More importantly, major complications were not associated with worse overall survival, local recurrence, or distant metastases [56]. 

Nizri et al. performed a single-institution study comparing patients who underwent a primary RPS resection (78 patients) and those who underwent a recurrent RPS resection (76 patients). They found that the recurrent RPS cohort had a lower resected organ score and transfusion requirement. There was no difference between postoperative complications and 30-day mortality between the two cohorts. Resected organ score and transfusion requirements were both associated with severe postoperative complications (Clavien-Dindo ≥ 3) in the recurrent RPS cohort [57].

## 6. Conclusions

To reduce local recurrence and improve overall survival, current guidelines for patients with RPS recommend complete resection of all macro and microscopic disease. Due to the anatomic constraints of the retroperitoneum and the diverse location from which these tumors can arise, this often requires a multi-visceral resection. With more extensive, compartmental resections, there is theoretical concern for increased surgical complications, but this remains controversial. Studies have demonstrated that even in the absence of gross invasion of the tumor into neighboring organs, the intimate relationship between the tumor and structures such as the mesentery or renal capsule often results in the microscopic invasion on final pathology. Since patients with RPS can achieve prolonged survival after surgery, it is unclear if the improvement in local control afforded by compartmental resections impacts overall survival. As such, keen clinical judgment by experienced sarcoma surgeons is of utmost importance to ensuring optimal oncologic outcomes while minimizing operative morbidity and mortality. Strategies to improve outcomes include patient optimization through preoperative nutrition and pre-habilitation programs, referral to high-volume sarcoma centers, and participation in postoperative enhanced recovery protocols.
